# Defective sarcoplasmic reticulum–mitochondria calcium exchange in aged mouse myocardium

**DOI:** 10.1038/cddis.2014.526

**Published:** 2014-12-18

**Authors:** C Fernandez-Sanz, M Ruiz-Meana, E Miro-Casas, E Nuñez, J Castellano, M Loureiro, I Barba, M Poncelas, A Rodriguez-Sinovas, J Vázquez, D Garcia-Dorado

**Affiliations:** 1 Cardiología, Hospital Universitari Vall d'Hebron, Institut de Recerca, Universitat Autònoma de Barcelona, Barcelona, Spain; 2 Centro Nacional de Investigaciones Cardiovasculares, Madrid, Spain

## Abstract

Mitochondrial alterations are critically involved in increased vulnerability to disease during aging. We investigated the contribution of mitochondria–sarcoplasmic reticulum (SR) communication in cardiomyocyte functional alterations during aging. Heart function (echocardiography) and ATP/phosphocreatine (NMR spectroscopy) were preserved in hearts from old mice (>20 months) with respect to young mice (5–6 months). Mitochondrial membrane potential and resting O_2_ consumption were similar in mitochondria from young and old hearts. However, maximal ADP-stimulated O_2_ consumption was specifically reduced in interfibrillar mitochondria from aged hearts. Second generation proteomics disclosed an increased mitochondrial protein oxidation in advanced age. Because energy production and oxidative status are regulated by mitochondrial Ca^2+^, we investigated the effect of age on mitochondrial Ca^2+^ uptake. Although no age-dependent differences were found in Ca^2+^ uptake kinetics in isolated mitochondria, mitochondrial Ca^2+^ uptake secondary to SR Ca^2+^ release was significantly reduced in cardiomyocytes from old hearts, and this effect was associated with decreased NAD(P)H regeneration and increased mitochondrial ROS upon increased contractile activity. Immunofluorescence and proximity ligation assay identified the defective communication between mitochondrial voltage-dependent anion channel and SR ryanodine receptor (RyR) in cardiomyocytes from aged hearts associated with altered Ca^2+^ handling. Age-dependent alterations in SR Ca^2+^ transfer to mitochondria and in Ca^2+^ handling could be reproduced in cardiomyoctes from young hearts after interorganelle disruption with colchicine, at concentrations that had no effect in aged cardiomyocytes or isolated mitochondria. Thus, defective SR–mitochondria communication underlies inefficient interorganelle Ca^2+^ exchange that contributes to energy demand/supply mistmach and oxidative stress in the aged heart.

Age is the main independent risk factor for cardiovascular morbidity and mortality.^1^ It increases heart vulnerability to cardiac diseases as well as the severity of their clinical manifestations, and reduces the efficacy of cardioprotective interventions.^2^ At the cellular level, some of the structural and functional age-dependent changes resemble those of failing cardiac myocytes.^3, 4^ Specifically, disturbed Ca^2+^ homeostasis and excitation–contraction coupling,^5^ as well as deficient mitochondrial energetics^6^ and excessive ROS production,^7^ have been consistently reported in senescent cardiomyocytes. These subcellular alterations likely contribute to the reduced adaptive capacity to stress (exercise, *β*-adrenergic stimulation) and increased vulnerability to disease of the aged hearts.

In cardiac cells, electrochemical coupling and metabolic adaptations are based upon the coordination between sarcoplasmic reticulum (SR) and mitochondria tightly interconnected forming an interface to support local ionic exchange and signal transduction in a beat-to-beat basis.^8^ This privileged interorganelle communication facilitates mitochondrial ATP transport for SR Ca^2+^ cycling and ensures energy replenishment by reciprocal Ca^2+^ and ADP exchange. Ca^2+^ is taken up by mitochondria using a low-affinity uniporter whose activity is driven by the elevated Ca^2+^ concentration in the microenvironment present around ryanodine receptors (RyR).^9^ Indeed, the kinetics of mitochondrial Ca^2+^ uptake is more dependent on the concentration of Ca^2+^ at the SR–mitochondria contact points than on bulk cytosolic Ca^2+^ concentration.^8^ Mitochondrial Ca^2+^ uptake allows energy supply–demand matching through the activation of Krebs cycle dehydrogenases and electron transport chain activity, and at the same time it regulates the regeneration of Krebs-coupled antioxidative defenses (NAD(P)H).^10^

Defective SR–mitochondria cross talk has been causally linked to the abnormal mitochondrial Ca^2+^ uptake in failing hearts and may underlie their increased oxidative stress.^11^ Also, in diabetic cardiomyopathy, intracellular Ca^2+^ overload and depletion of energy stores appear to develop as a consequence of sequential SR–mitochondria dysfunction.^12^ Atrial fibrillation has been associated with an increased fusion of mitochondria and a subsequent increased colocalization of giant mitochondria with SR, a subcellular remodeling process that contributes to the perpetuation of the arrhythmia.^13^ Because mitochondria are highly dynamic structures, some molecular links have been proposed to provide a stable physical interorganelle bridge^14, 15^ while others appear to facilitate direct tunneling of Ca^2+^ and other signaling mediators.^16^ In the present study, we hypothesized that aging may negatively impact on mitochondria–SR communication by mechanisms involving defective Ca^2+^ transmission, and we identified reduced physical interaction between RyR and mitochondrial voltage-dependent anion channel (VDAC) as the main responsible of this effect.

## Results

### Aging phenotype

**Figure 1:**
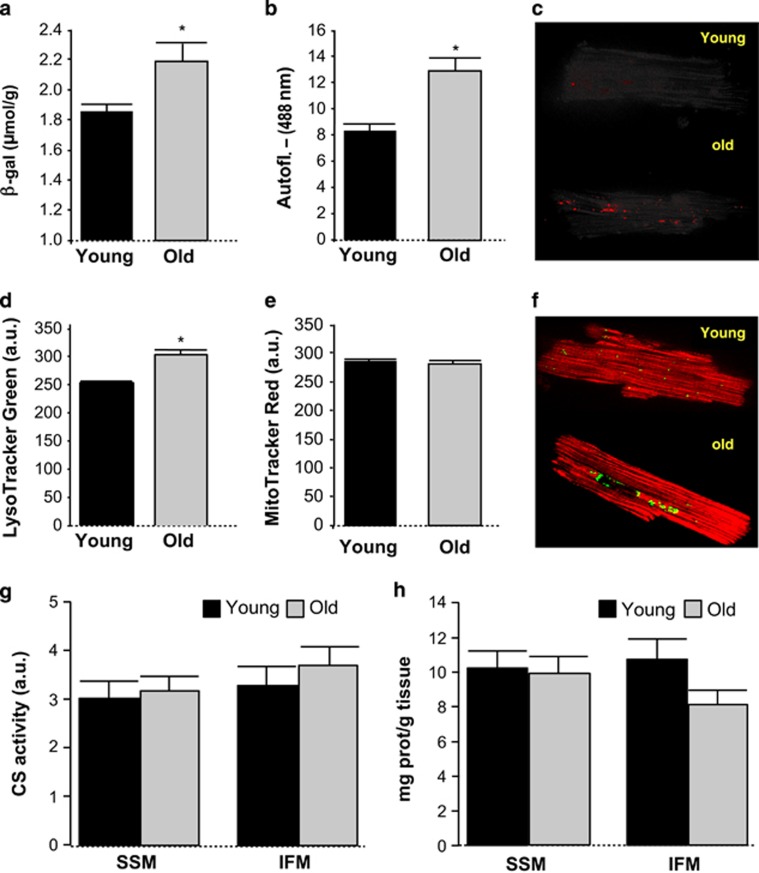
Effect of advanced age on (**a**) *β*-galactosidase, (**b** and **c**) lipofuscin autofluorescence, (**d** and **f**, green color) lysosome vesicles, (**e** and **f**, red color) mitochondrial pool labeling in intact mouse cardiomyocytes, (**g**) citrate synthase (CS) activity in isolated heart mitochondria and (**h**) mitochondrial yield determined as mitochondrial protein with respect to total cardiac protein in subsarcolemmal (SSM) and interfibrillar (IFM) mitochondria isolated from mouse hearts. Mean±S.E.M. from *n*=14–25 replicates (5–10 hearts)

Echocardiographic analysis showed thinner ventricular wall and a trend toward increased LV end-diastolic volume in old mice, as well as a nonsignificant trend toward reduced ejection fraction (Table 1). Isolated cardiomyocytes from aged hearts displayed an increase in lysosome vesicles, lipofuscin pigment and *β*-galactosidase (*β*-gal) activity, as hallmarks of cell senescence (Figure 1). There were no age-dependent differences in mitochondrial pool, as quantified by Mitotracker red staining, quantification of citrate synthase (CS) activity and total cardiac mitochondrial yield (Figure 1).

### Effect of aging on energy metabolism and mitochondrial respiration

**Figure 2:**
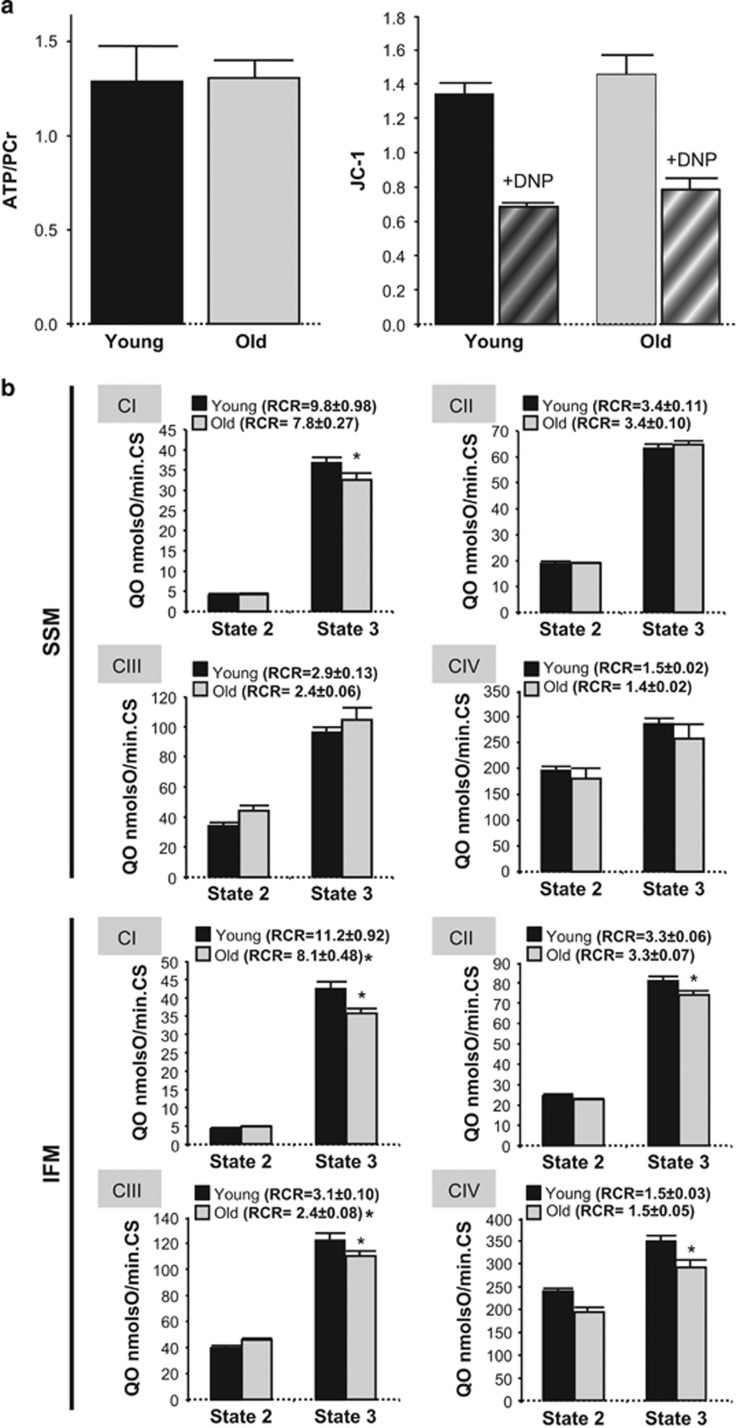
(**a**) ATP/PCr in intact hearts from young and old mice, quantified by NMR spectroscopy (left panel; *n*=3 hearts). Mitochondrial membrane potential (JC-1 ratio fluorescence) in intact cardiac myocytes from young and old mouse hearts under resting contidions and after induction of maximal mitochondrial depolarization with DNP (right panel). (*n*=14–26 cardiomyocytes per group, five hearts). (**b**) Resting O_2_ consumption (state-2) and ADP-stimulated O_2_ consumption (state-3) in subsarcolemmal and interfibrillar mitochondria from young (<6 months) and old (>20 months) mouse hearts, mediated by substrates of complexes 1–4, normalized by citrate synthase (CS) activity. RCR, respiratory control rate for each respiratory complex (state-3/state-2). Mean±S.E.M. from *n*=14–28 replicates (5–10 hearts)

Myocardial ATP/phosphocreatine ratio was similar in hearts from both age groups. Advanced age did not induce mitochondrial membrane depolarization under resting conditions as quantified in JC-1 loaded isolated cardiomyocytes (Figure 2a). Both O_2_ consumption at rest (state-2) and after ADP-stimulation (state-3) were largely preserved in subsarcolemmal mitochondria from aged mouse hearts, independently of the substrates used to feed the respiratory complexes, except a slight decrease in complex 1-mediated state-3 respiration, without changes in respiratory control rate (RCR; Figure 2b). By contrast, interfibrillar mitochondria (IFM) from aged hearts displayed a depression of ADP-stimulated O_2_ consumption for any substrate used to feed respiratory complexes, with reduced RCR dependent on complexes 1 and 3 (Figure 2b). These data indicate an altered capacity of IFM from aged hearts to respond to maximal stimulation, despite of preserved respiration under resting conditions.

### Aging is associated with altered SR RyR gating properties

**Figure 3:**
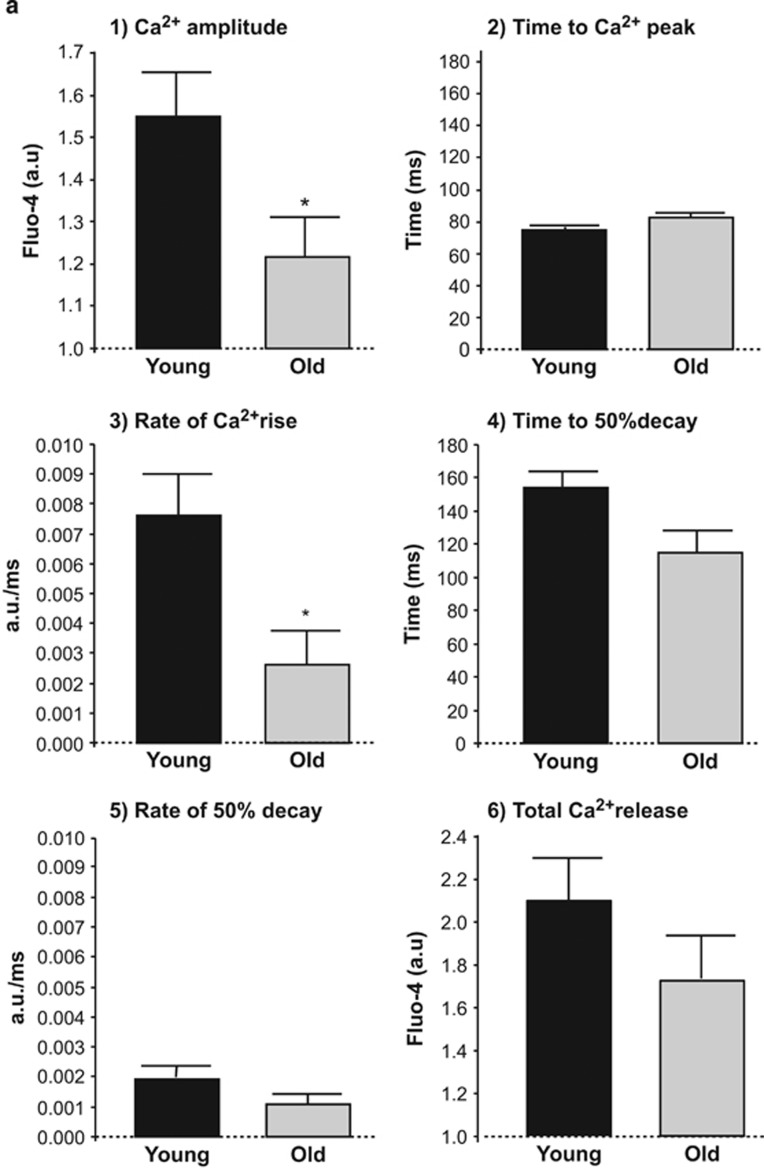

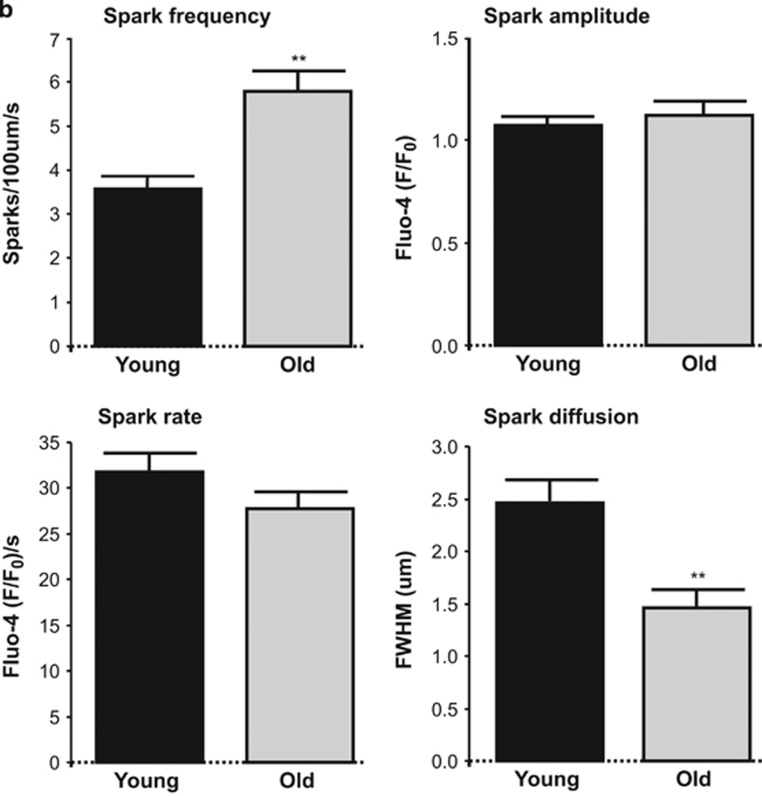
(**a**) Effect of aging on Ca^2+^ transient amplitude (panel-1), and SR Ca^2+^ release/uptake kinetics in field-stimulated (1 Hz) fluo-4 loaded cardiac myocytes from young and old mouse hearts (panels-2 to 5) and in total SR Ca^2+^ content after caffeine stimulation (panel-6). *, *P*<0.05 with respect to young, *n*=9–12 cardiomyocytes per group (six hearts). (**b**) Spontaneous spark frequency, amplitude, rate and diffusion in quiescent fluo-4 loaded cardiomyocytes from young and old mouse hearts. FWHM, full width at half maximum (μm). **, *P*<0.001 with respect to young. Data represent mean±S.E.M. from 1600 sparks (34 cardiomyocytes, four hearts)

Field stimulation of cardiomyocytes disclosed a reduction in the amplitude of SR Ca^2+^ transients and a decreased rate of Ca^2+^ rise in cardiomyocytes from old hearts, without differences in the time to peak or time to 50% decay (Figure 3a). SR Ca^2+^ content, quantified as maximal caffeine-induced Ca^2+^ release, was similar in cardiomyocytes from both age groups (Figure 3a). In non-stimulated cardiomyocytes, aging was associated with a significant increase in the frequency of spontaneous SR Ca^2+^ sparks with decreased Ca^2+^ diffusion, without changes in their rate or amplitude (Figure 3b). These data indicate altered RyR gating properties in old cardiomyocytes.

### Advanced age depresses SR–mitochondria calcium transfer and NAD(P)H regeneration

**Figure 4:**
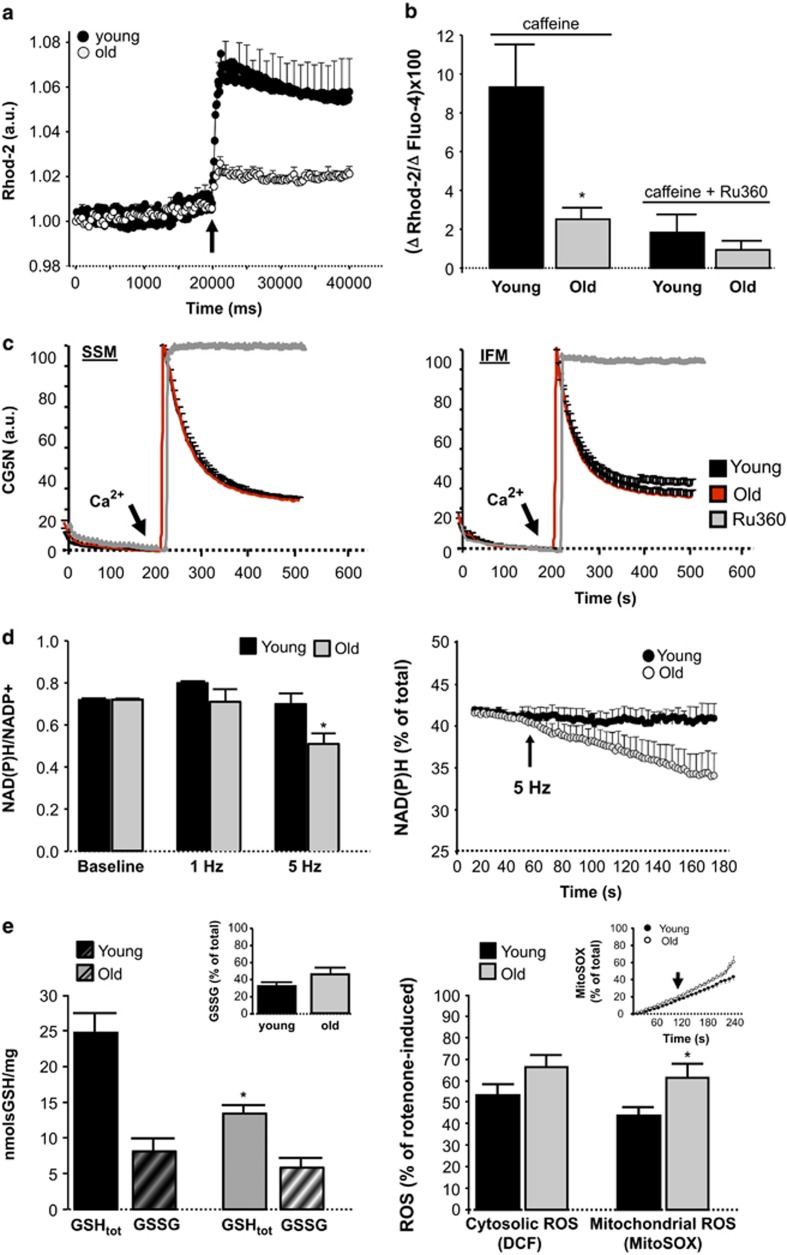
(**a**) Mitochondrial Ca^2+^ uptake throughout time in response to SR Ca^2+^ release (10 mmol/l caffeine, arrow) in digitonin-permeabilized rhod-2 loaded cardiac myocytes from old and young mouse hearts. *N*=8–11 cardiomyocytes per group (*n*=5 hearts). (**b**) Maximal mitochondrial Ca^2+^ uptake (rhod-2) normalized by maximal SR Ca^2+^ release (fluo-4) in young and old permeabilized mouse cardiomyocytes. Addition of 10 *μ*mol/l Ru360 (a specific blocker of the mitochondrial Ca^2+^ uniporter) prevented caffeine-induced mitochondrial Ca^2+^ uptake in both groups of ages. *, *P*<0.05 with respect to young. *n*=7–11 cardiomyocytes per group (five hearts). (**c**) Absence of age-dependent differences in the *in vitro* mitochondrial Ca^2+^ uptake kinetics (CG5N fluorescence), when exposing isolated subsarcolemmal (SSM) and interfibrillar (IFM) cardiac mitochondria to an external Ca^2+^ pulse of 30 *μ*mol/l (arrow). Addition of 10 *μ*mol/l Ru360 prevented mitochondrial Ca^2+^ uptake. Mean±S.E.M. of four replicates per group (six hearts). (**d**) NAD(P)H/NAD(P)+ ratio 2 min after electrical stimulation at 1 Hz and 5 Hz to induce high contractile activity in intact cardiomyocytes from young and old mouse hearts (left panel), and kinetics of the NAD(P)H consumption in its reduced form, expressed with respect to total cell NADPH, during 5 Hz stimulation (right panel). Data represent mean±S.E.M. (10–19 cardiomyocytes per group, nine hearts). (**e**) Left panel: total glutathione (GSHtot) and oxidized glutathione levels (GSSG) in myocardial tissue of young and old mice. The inset shows the fraction of oxidized glutathione with respect to total one; Right panel: cytosolic and mitochondrial ROS production 2 min after electrical stimulation at 5 Hz to induce high contractile activity in intact cardiomyoctes from young and old mouse hearts, as quantified by DCF and MitoSox fluorescence. The inset shows the kinetics of short-term mitochondrial ROS production during pacing (arrow points the onset of 5 Hz stimulation)

In digitonin-permeabilized cardiomyocytes from young hearts, induction of SR Ca^2+^ release with caffeine was followed by a rapid increase in mitochondrial Ca^2+^ uptake that was severely depressed in cardiomyocytes from old hearts (Figure 4a). Ca^2+^ uptake by mitochondria was dependent on mitochondrial Ca^2+^ uniporter and could be prevented by the specific inhibitor Ru360 (Figure 4b). However, this depression in mitochondrial Ca^2+^ uptake kinetics observed in intact cardiomyocytes of aged hearts could not be reproduced in isolated mitochondria exposed to an external Ca^2+^ pulse *in vitro* (Figure 4c). Field stimulation at 1 Hz did not result in net consumption of NAD(P)H in cardiomyocytes from any group of age, but rather induced a slight increase in young cells. However, acceleration of electrical pacing from 1 Hz to 5 Hz was associated with a decay in NAD(P)H/NAD(P)+ in cardiomyocytes from old hearts but not from young hearts, indicating an inefficient NAD(P)H regeneration (Figure 4d).

All together, these data suggest a defective Ca^2+^ transfer from SR to mitochondria, with concomitant reduced bioenergetic feedback response (NAD(P)H regeneration), in cardiomyocytes of aged hearts, despite normal mitochondrial Ca^2+^ uniporter activity.

### Aged cardiomyocytes have less glutathione and increased mitochondrial ROS production

Total glutathione (GSH) levels (GSHtot) quantified in myocardial tissue were significantly decreased in aging hearts (Figure 4e, left panel). The oxidized fraction of GSH (glutathione disulfide, GSSG) showed a trend toward an increase in the aging myocardium (Figure 4e, left panel). In isolated cardiomyocytes submitted to electrical stimulation, aging was associated with a significant increase in the short-term mitochondrial ROS production during high frequency pacing (5 Hz). There was a nonsignificant trend toward an increased cytosolic ROS levels in aging cardiomyocytes after high frequency pacing, but we cannot rule out that this is due to diffusion of mitochondrial ROS toward the cytosolic.

### Spatial proximity between mitochondria and SR decreases with aging

**Figure 5:**
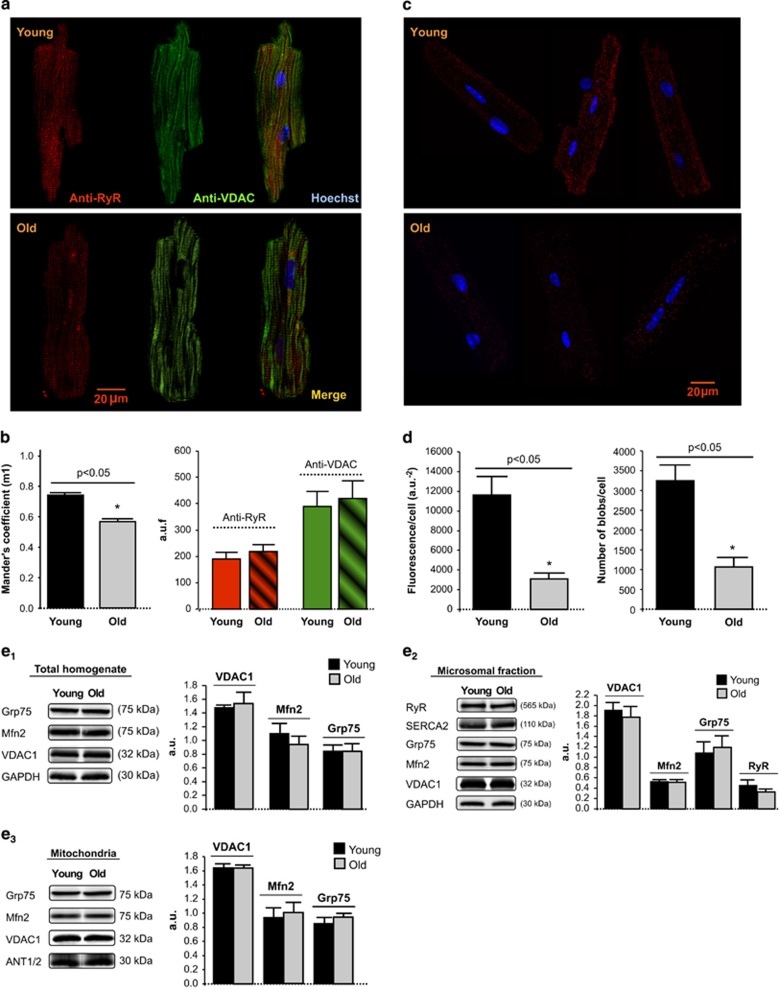
(**a**) Confocal fluorescent images of young and old mouse cardiomyocyte simultaneously labeled with anti-RyR (red), anti-VDAC (green) and Hoechst (blue) for visualization of SR, mitochondria and nuclei, respectively. (**b**) Effect of aging on RyR–VDAC spatial interaction, as quantified by Mander's coefficient (m1) analysis, expressed as the percentage of RyR – with respect to total RyR fluorescence – that overlaps with VDAC (left panel); in the right panel, total RyR and VDAC fluorescence. Mean±S.E.M. from 4 to 6 cardiomyocytes per group (four hearts). (**c**) Confocal fluorescent images of the RyR–VDAC interaction in different individual cardiomyocytes isolated from young and old mouse hearts, detected by proximity ligation assay (PLA). Positive cross-reactivity – reflecting an intermolecular distance of <40nm – is shown in red, nuclei are depicted in blue (Hoechst). (**d**) Aging was associated with a significant reduction in cell fluorescence resulting from RyR–VDAC cross-reactivity (left panel) and in the number of amplification spots (right panel), as quantified by PLA assay. Mean±S.E.M. of 1071–3250 blobs per group (15 cardiomyocytes, two hearts). (**e**) Western blot representative bands and quantification of the expression of proteins involved in SR and mitochondria Ca^2+^ transport and interorganelle communication in whole-heart homogenate, microsomal and mitochondrial fractions from young and old mice. Each protein of interest was normalized by the corresponding protein of reference as follows: in total homogenates, VDAC1/Grp75, Mfn2/GAPDH and Grp75/GAPDH; in microsomal fraction, Mfn2/VDAC1, VDAC1/Grp75, Grp75/GADPH and RyR/Grp75; in mitochondria, VDAC1/Grp75, Grp75/ANT1/2 and Mfn2/ANT1/2. Mean±S.E.M. of *n*=8 replicates from four hearts/group)

Simultaneous immunolabeling of permeabilized cardiomyocytes with anti-RyR and anti-VDAC disclosed a pattern of close proximity and similar spatial organization between SR and mitochondria (Figure 5a). Blind quantification of immunocolocalization degree by Mander's coefficient analysis revealed an age-dependent reduction of the fraction of RyR overlapping with VDAC (m1) without significant differences in either RyR or VDAC total fluorescences (Figure 5b). Proximity ligation assay (PLA), specifically addressed to quantify SR–mitochondria clusters of <40 nm distance, demonstrated an age-dependent reduction in the positive amplification fluorescent spots (Figures 5c and d). These data indicate a reduction in the fraction of SR RyR closely juxtaposed with mitochondrial VDAC.

**Figure 6:**
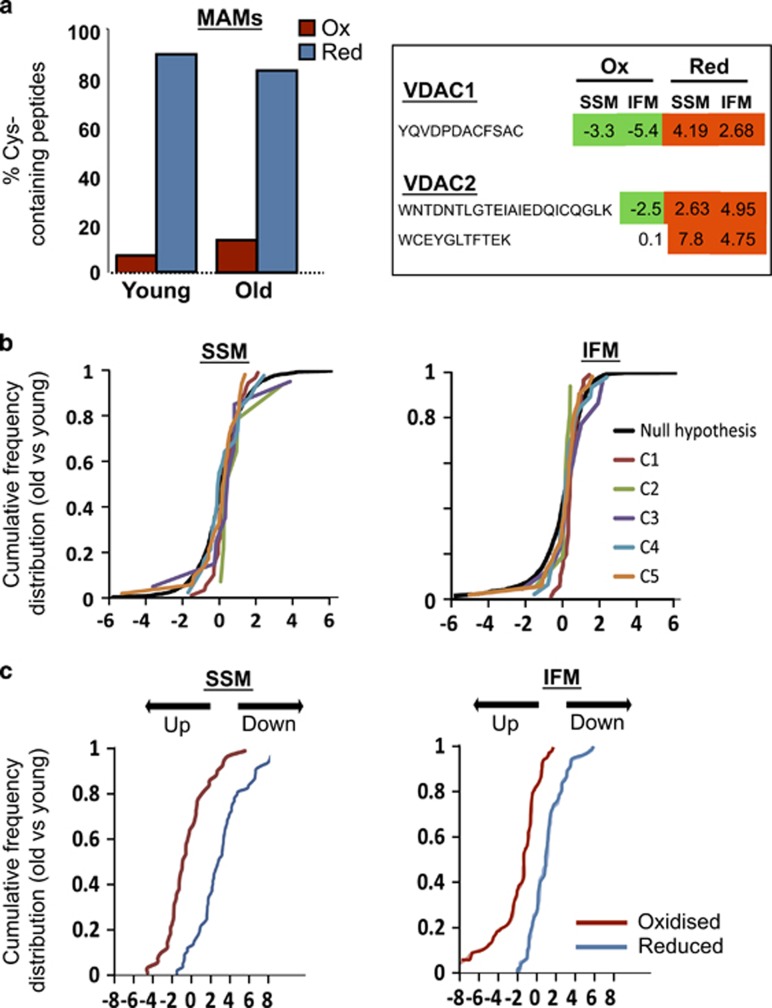
Effect of aging on: (**a**) quantitative redox proteomics using GELSILOX of proteins from mitochondria-associated membranes (MAMs; left) and of the Cys-containing peptides detected in mitochondrial VDAC proteins (right). The left graph represents the percentage of Cys-containing peptides identified in oxidized (red) or reduced (blue) forms. The numbers in the right table represent the standardized variable at the peptide level as in **b**; negative values indicate an increase (in green) and positive values a decrease (in red) in the peptides containing Cys in oxidized form (Ox) or in reduced form (Red) in mitochondrial VDAC proteins in hearts from old mice. (**b**) The abundance of mitochondrial respiratory complexes (1–5) in subsarcolemmal (SSM) and interfibrillar (IFM) mitochondria. Data are shown as cumulative distributions of the standardized variable at the protein level (i.e., corrected log2 ratios of proteins expressed in units of standard deviation) for all oxidative phosphorylation proteins. The black sigmoid is the theoretical null hypothesis distribution; a displacement toward the left indicates an increase in protein concentration. All the categories follow very closely the null hypothesis distribution, indicating that aging does not affect the abundance of mitochondrial respiratory proteins. (**c**) Alterations in the abundance of oxidized (red) and reduced (blue) Cys-containing peptides in SSM and IFM from young and old mice hearts. Peptides containing Cys residues in different oxidation states were quantified using the GELSILOX method. The sigmoid curves represent the cumulative distribution of the standardized variable at the peptide level (i.e. corrected log2-ratios of peptides expressed in units of S.D.), for all peptides containing either oxidized or reduced Cys sites that belong to proteins from OxPhos complexes

The effect of age on SR–mitochondria dissociation was not explained by a reduced expression of the main proteins postulated to tether both organelles. Western blot quantification of mitofusin-2, VDAC1, RyR2 and Grp75 in total cardiac homogenates, microsomal fractions and mitochondria showed no differences between both groups of age (Figure 5e). These results were consistent with those of quantitative proteomics analysis (not shown). However, individual peptide quantification by GELSILOX of the same data revealed an increase in the proportion of peptides containing oxidized Cys detected in mitochondrial VDAC and a concomitant reduction in the same peptides containing the reduced Cys form in both subsarcolemmal (SSM) and IFM from old mouse hearts, suggesting that aging induces oxidative damage in this protein. Consistently, proteins from mitochondria-associated membrane (MAM) fraction displayed a trend toward and increased oxidative state (Figure 6a). These data are compatible with the existance of an oxidized microenvironment around SR–mitochondria contact points, despite normal protein expression.

### Increased oxidation of mitochondrial respiratory proteins in aged hearts

High-throughput quantitative proteomics followed by systems biology analysis indicated that there were no age-dependent changes in relative abundance of proteins of any of the five oxidative phosphorylation complexes in SSM or IFM (Figure 6b and Supplementary Table 1). However, GELSILOX analysis revealed that aging was associated with an increased proportion of mitochondrial peptides containing oxidized Cys and a decrease in those containing reduced Cys in proteins of the Oxphos complexes (Figure 6c and Supplementary Table 2).

### Disruption of SR–mitochondria connection in young cardiomyocytes mimicks age-induced alterations in calcium handling

**Figure 7:**
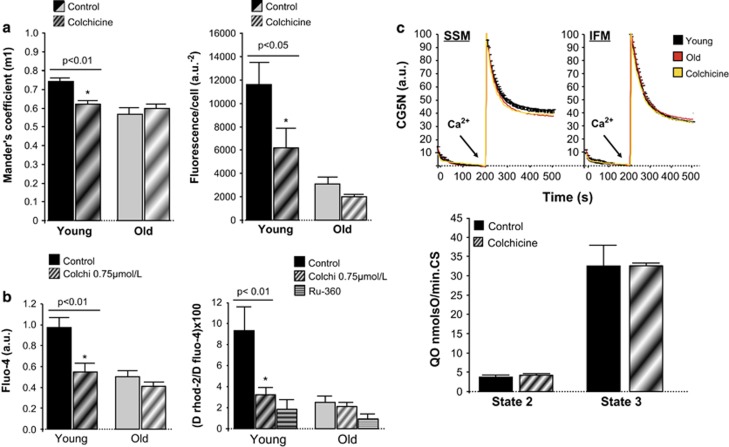
Effect of 0.75 *μ*mol/l colchicine on: (**a**) RyR–VDAC spatial interaction quantified by Mander's coefficient (m1) analysis, indicating a reduction of the percentage of RyR (with respect to total RyR) that overlaps with VDAC in young cardiac myocytes, without effect in old cardiomyoyctes (left panel); and RyR–VDAC positive cross-reactivity detected by proximity ligation assay (PLA; right panel; *n*=4–6). (**b**) The amplitude of Ca^2+^ transients in fluo-4 loaded cardiomyocytes submitted to field-stimulation (left panel), and mitochondrial Ca^2+^ uptake in response to SR Ca^2+^ release in digitonin-permeabilized cardiac myocytes (right panel). Ru360 10 *μ*mol/l was used to specifically inhibit mitochondrial Ca^2+^ uniporter (*n*=6–11). (**c**) *In vitro* mitochondrial Ca^2+^ uptake (CG5N fluorescence) in isolated subsarcolemmal (SSM) and interfibrillar (IFM) mitochondria from young mouse hearts with or without colchicine (yellow and black), and from old mouse hearts (red), exposed to an external 30 *μ*mol/l Ca^2+^ pulse (arrow; top panels), complex 11-mediated O_2_ consumption (state-2) and ADP-stimulated O_2_ consumption (state-3) in SSM from young mouse hearts, normalized by citrate synthase activity, in the absence (control) or in the presence of colchicine (bottom panel). Mean±S.E.M. of four replicates per group (six hearts)

Addition of 0.75 *μ*mol/l colchicine to intact cardiomyocytes induced a partial disruption of SR–mitochondria interaction, confirmed by both decreased RyR–VDAC immunocolocalization and reduced number of amplification clusters in PLA assay (Figure 7a). The effect of this intervention was significantly less pronounced in cardiomyocytes of aged hearts. In cardiomyocytes from young mice, colchicine induced a parallel depression in the amplitude of SR Ca^2+^ transients in intact cells and decreased mitochondrial Ca^2+^ uptake secondary to SR Ca^2+^ transfer (caffeine) in permeabilized cells, but it had only little nonsignificant effect in cardiomyocytes from old mice (Figure 7b). These data indicate that partial pharmacological disruption of SR–mitochondrial communication induces Ca^2+^ handling alterations that resemble those that are constitutively present in old cardiomyocytes. Nevertheless, colchicine did not have any effect on Ca^2+^ uptake kinetics or respiration efficiency in isolated mitochondria of any group of age (Figure 7c).

## Discussion

The results of the present study indicate that SR–mitochondria communication is altered in aged cardiomyocytes, as manifested by reduced physical interaction among RyR and VDAC and depressed local Ca^2+^ transfer from SR to mitochondria, despite normal mitochondrial Ca^2+^ uniporter activity and SR Ca^2+^ content. As a consequence of reduced Ca^2+^ exchange, mitochondrial regeneration of the electron donor NADH and the antioxidant NADPH is depressed in old cardiomyocytes when exposed to increased frequency of pacing, reflecting an uncoupled bioenergetic feedback response, and mitochondrial ROS production is concomitantly increased under these conditions. IFM mitochondria – the population of mitochondria closely juxtaposed with SR – displayed reduced maximal O_2_ consumption and altered RCR. GSH levels are reduced in aging myocardium and differential redox proteomics using GELSILOX identified an age-dependent increase in Cys oxidation of mitochondrial VDAC and a trend toward an overall increase in the oxidative state of MAM proteins. This microenvironmental oxidation at the mitochondria–SR contact points was associated with abnormal RyR gating properties in SR from aged cardiomyocytes. These data identify a novel subcellular mechanism of functional decline during aging that could help to the development of cardioprotective strategies specifically addressed to the aged heart.

### Disruption of SR–mitochondria spatial relationship during aging

In cardiac myocytes, SR is surrounded by a dynamic network of mitochondria extending along myofibril length.^17^ The positioning of mitochondria is supported by mitochondrial anchorage to cytoskeleton and SR through proteolysis-sensitive electron-dense tethering structures.^18^ Several molecular entities have been proposed to bridge the terminal cisternae of the SR or endoplasmic reticulum with mitochondria, as mitofusins 1 and 2,^15^ the complex formed by RyR/IP3 and the outer mitochondrial membrane VDAC connected through the cytosolic chaperone Grp75,^16^ and other proteins related with apoptotic pathways,^14^ but their exact contribution to physical or chemical coupling or whether they may be redundant remains controversial. In cardiac cells, mitochondrial Ca^2+^ uptake secondary to SR Ca^2+^ release, essential for short and long-term metabolic adaptations, gives rise to locally restricted high calcium cell domains.^9^ To accomplish this, up to 90% of the Ca^2+^ release units of ventricular SR are close to mitochondria, with an estimated interorganelle surface distance of 37 nm.^19^ Our results demonstrate a partially disrupted VDAC and RyR interaction in ventricular cardiomyocytes from aging hearts, determined by two independent techniques, immunocolocalization and PLA. Mander's coefficient analysis of SR and mitochondria immunolabelling indicates that, while most RyR fraction (>75%) overlaps with mitochondrial VDAC in young cardiomyocytes, this interaction is reduced to <60% in old cardiomyocytes. PLA, specifically addressed to detect those SR–mitochondria native clusters in which the spatial distance between RyR and VDAC is ⩽40 nm, confirmed the dissociating effect of age at this subcellar level.

Altered RyR–VDAC interaction in old hearts cannot be explained by a decrease in the expression of these proteins, because the amount of RyR and VDAC, as quantified by immunofluorescence labeling, western blot and proteomics, was similar in both groups of ages. The expression of other proteins potentially involved in SR–mitochondria bridging, like mitofusin-2 and the cytosolic chaperone Grp75, also remained preserved in old hearts at the different subcellular fractions. Because mitochondria are the major producers of superoxide, it is reasonable to expect that sustained mitochondrial ROS production derived from respiratory activity locally impacts on redox-sensitive proteins at the subcellular microdomain. Indeed, our experiments show increased mitochondrial ROS production in aging cardiomyocytes submitted to increased contractile activity and redox microdomains have been described at the interface between SR and mitochondria, in which NADPH oxidases contribute to ROS generation.^20^ Our proteomics data point to an increased overall oxidation of the MAM fraction, the subcellular proteinaceous SR–mitochondria tethering structure involved in intracellular Ca^2+^ signaling and bioenergetics regulation,^21^ as well as an increased oxidation of mitochondrial VDAC. However, the efficiency of our proteomics approach to specifically obtain Cys-containing peptides within RyR molecules is too low to get a reliable quantification of their redox state, probably due to the deep localization of the thiol sites in the receptor. RyR channels contain multiple, potentially redox-sensitive cysteine residues, and cysteine thiol oxidation appears to increase RyR channel activity.^22^ Our functional data on SR Ca^2+^ handling are fully consistent with an oxidation-induced alteration of the RyR gating properties, manifested by a substantial increase in spontaneous Ca^2+^ spark frequency and altered morphology (reduced Ca^2+^ diffusion pattern) and decreased Ca^2+^ transient amplitude in the aged cardiomyocytes, despite no significant alteration in total SR Ca^2+^ content. These SR Ca^2+^ abnormalities have been attributed to increased frequency of RyR opening events of less unitary duration and have been described in oxidative environments like those present in failing and aging hearts.^5, 23, 24^ It remains to be elucidated what type of conformational changes may occur in RyR proteins exposed to oxidative stress and how these changes may potentially modify their interaction with other proteins.

### Defective SR–mitochondria communication impacts on local calcium handling

Our experiments demonstrate that old cardiomyocytes exhibit a depressed mitochondrial Ca^2+^ uptake that is secondary to reduced Ca^2+^ transfer from SR. This concept is supported by several observations: (1) it is specifically manifested in response to RyR stimulation with caffeine; (2) it takes place in digitonin-permeabilized cardiomyocytes, in which the contribution of sarcolemma and cytosol to mitochondrial Ca^2+^ handling is absent; (3) mitochondrial Ca^2+^ uptake is strictly normal when exposing isolated mitochondria to external Ca^2+^ *in vitro*. Our data also indicate that reduced SR Ca^2+^ transfer in aged cardiomyocytes results in defective NAD(P)H regeneration when cells are submitted to increased contractile activity (high-rate pacing), indicating an altered bioenergetic feedback response. Moreover, impaired mitochondrial Ca^2+^ uptake in aged cardiomyocytes can be reproduced in young cells after pharmacological disruption of SR–mitochondrial physical interaction with colchicine. Of note, this disrupting maneuvre does not have any effect on mitochondrial Ca^2+^ uniporter activity or O_2_ consumption, which could reduce mitochondrial Ca^2+^ uptake by mechanisms independent of interorganelle communication.

An increasing number of evidences support the view that the coordination between mitochondrial ATP supply and mechanical activity demand is highly dependent on an efficient Ca^2+^ transfer from SR to mitochondria,^15, 25^ and that altered SR–mitochondria Ca^2+^ exchange may underlie the pathophysiology of several cardiac diseases.^26^ In an acute setting, like the first phase of myocardial reperfusion, rapid and cyclic SR-induced Ca^2+^ oscillations – in a hyperoxidative cellular milieu – trigger mitochondrial permeabilization, hypercontracture and cell death.^27, 28^ In this context, genetic or pharmacological inhibition of SR–mitochondria Ca^2+^ transfer have been demonstrated to have beneficial effects on cell survival.^29^ By contrast, chronically insufficient mitochondrial Ca^2+^ uptake due to defective SR–mitochondrial spatial organization contributes to oxidative stress and energy deficiency.^15^ This is because Ca^2+^ released by RyR is partially transferred to adjacent mitochondria, where it activates Ca^2+^-sensitive Krebs dehydrogenases and other downstream enzymes involved in energy production, like F1–F0 ATPase and adenine nucleotide translocase, as well as antioxidant pool regeneration.^30, 31, 32^ Therefore, ablation of mitofusin-2 not only disrupts SR–mitochondrial cytoarchitecture but has been demonstrated to alter Ca^2+^ transients and mitochondrial Ca^2+^ uptake and to induce bioenergetic–redox mismatch in cardiac myocytes submitted to *β*-adrenergic stimulation.^15^ In a recent three-dimensional integrated cardiomyocyte computational model,^33^ increasing SR–mitochondria distance from 50 nm to 200 nm depressed mitochondrial Ca^2+^ uptake by 17% and NADH generation by 11% when pacing rates were switched from 0.5 to 2 Hz.^33^ In our experiments, depressed NAD(P)H regeneration in response to pacing stress occurred only in cardiomyocytes from aged hearts, indicating impaired bioenergetic feedback response secondary to inadequate mitochondrial Ca^2+^ uptake, and was associated with increased ROS production at the mitochondrial compartment. This mechanism may aggravate the age-associated excess of toxic ROS in a vicious cycle. Importantly, the impact of aging on mitochondrial maximal O_2_ consumption capacity and RCR was specifically manifested in IFM. This finding is consistent with previous observations suggesting a greater susceptibility of IFM to aging ^34, 35^ and should be put in the context that this mitochondrial population represents the most important fraction of total cell mitochondria closely juxtaposed with SR, although other mitochondria may contact with RyR2.^36^ Remarkably, resting O_2_ consumption, ATP/PCr and Δ*Ψ*m are preserved in old hearts, indicating that age-dependent mitochondrial energy deficiency develops only under stressful conditions (ADP-induced maximal O_2_ consumption and increased contractile activity) and involves the mitochondrial bioenergetics feedback response (NADPH regeneration and RCR). This bioenergetic inefficiency cannot be attributed to reduced mitochondrial mass, which seems to remain preserverd in aging mouse cardiomyocytes as quantified by specific staining with Mitotracker red, total cardiac mitochondrial yield quantification and CS activity measurement.

Aging is characterized by augmented entropy, in which random and stochastic episodes not genetically controlled result in biologically defective molecules. Some covalent modifications – that is, oxidation, glycosilation, phosphorylation, conformational changes and genomic mutations – eventually exceed cell repair capability.^37^ An insidious increase in the oxidation status has been consistently observed during senescence.^3, 4^ Our data confirm that mitochondrial respiratory complexes are overoxidized in the aged cardiomyocytes. This post-translational damage can reflect excessive ROS generation, decreased antioxidant capacity or reduced degradation of oxidized proteins. Recent evidences have suggested the defective mitochondrial Ca^2+^ uptake is one of the mechanisms involved in impaired antioxidant regeneration in failing cardiomyocytes.^11, 38^

**Figure 8:**
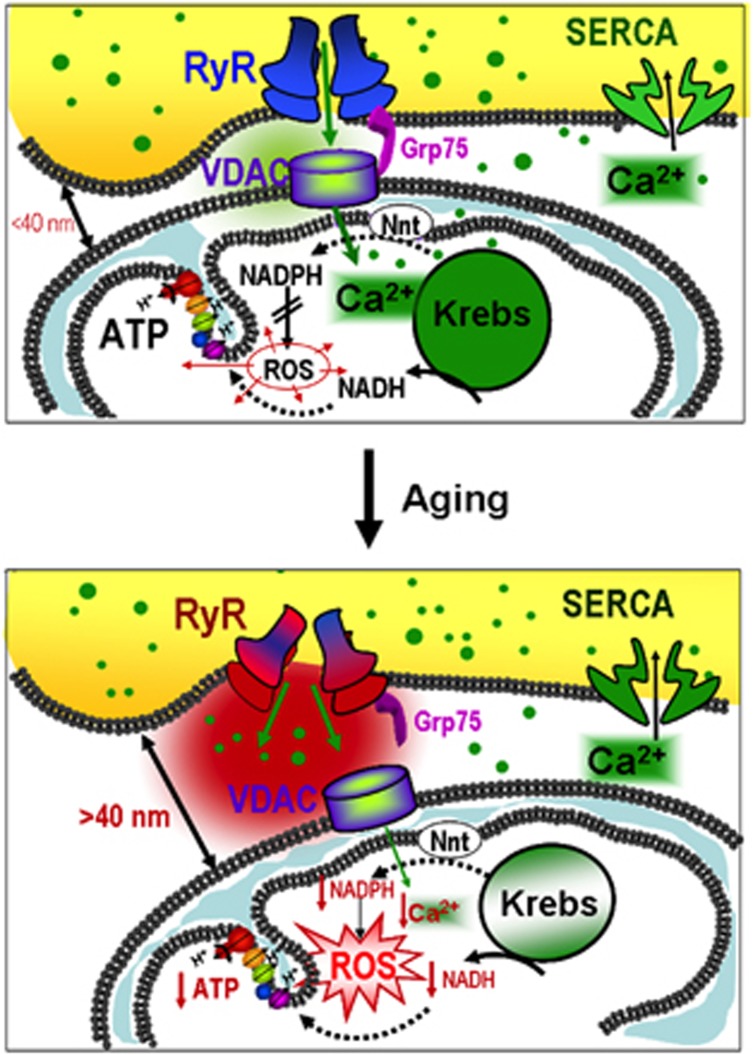
Schematic representation of the proposed mechanism by which aging induces SR–mitochondria disruption in cardiomyocytes

In conclusion, we propose a pathophysiological mechanism summarized in Figure 8, in which mitochondrial ROS derived from oxidative phosphorylation locally impacts on different protein targets, including electron transport chain and bridging proteins of the interorganelle space. Oxidative damage does not have significant consequences on mitochondrial energy production in old hearts under resting conditions but eventually disrupts the intimate connection between mitochondria and SR. The resulting alteration in RyR gating properties and SR Ca^2+^ handling may, however, amplify cell damage in a positive feedback, as deficient Ca^2+^ transfer from RyR to adjacent mitochondria further increases oxidative damage (impairing NADPH regeneration) and ultimately leads to inefficient energy production.

## Materials and Methods

Young adult (5–6 months) and old (>20 months) C57BL/6 mice were used for *in situ* functional analysis (echochardiography) and for the obtention of myocardial tissue, isolated cardiomyocytes and mitochondria. Animal handling was approved by Research Commission on Ethics of the Hospital Vall d'Hebron. All procedures conformed to EU Directive 2010/63EU and Recommendation 2007/526/EC regarding the protection of animals used for experimental and other scientific purposes, enforced in Spanish law under Real Decreto 1201/2005.

### Transthoracic Echocardiographic Analysis

Echocardiographic measurements were performerd in young and old mice under light anesthesia (isofluorane 0.5–1%) with a Vivid-Q portable ultrasound system using a ILS 12 MHz transducer (GE Healthcare, Wauwatosa, WI, USA). Conventional parameters (ejection fraction of the left ventricle (LVEF), left ventricular end-diastolic diameter (LVEDD), left ventricular end-systolic diameter (LVESD), septum wall thickness (SWT) and posterior wall thickness (PWT)) were measured in M-mode recordings at the level of the papillary muscles. Left ventricular fractional shortening was calculated as ((LVEDD–LVESD)/LVEDD) × 100. Left ventricular mass was calculated as 0.8x(1.04 × (LVEDD+ PWT+SWT)^3^ – (LVEDD)^3^)+0.6.

### NMR spectroscopy

Myocardial energetic status was evaluated in Langendorf-perfused intact mouse hearts by NMR spectroscopy. ATP/PCr was measured as described.^39^ Spectra were acquired in a 9.4T vertical magnet interfaced to a Bruker AVANCE 400 spectrometer (Billerica, MA, USA) tuned to 161.97 MHz; each spectra consisted in the accumulation of 400 scans and lasted 14 min. The areas of Pcr and *γ*-ATP peaks were measured by deconvolution.

### Isolation of heart mitochondria

SSM and IFM cardiac mitochondria were isolated by differential centrifugation from mouse hearts as originally described by Palmer *et al.*^40^ Ventricular tissue was minced in cold 'buffer A', containing (in mmol/l): 290 sucrose, 5 MOPS at pH 7.4, 2 EGTA and 0.2% deffated albumin. Minced tissue was mildly homogenized using a Potter–Elvehjem device. Homogenates were centrifuged at 750 × *g* for 5 min. Pellets and supernatants were processed independently to isolate different mitochondrial pools. For SSM mitochondria fraction, supernatants were centrifuged at 5000 × *g* for 5 min. For IFM fraction, the resulting pellets were resuspended in cold 'buffer B', containing (in mmol/l): 100 KCl, 5 MOPS at pH 7.4, 2 EGTA and 0.2% deffated albumin with proteinase K (P2308, Sigma, Munich, Germany) at 2 mg/g wet weight, and quickly homogenized. Homogenates were centrifuged at 750 × *g* for 5 min. Supernatants were subjected to a subsequent centrifugation at 5000 × *g* for 5 min to obtain IFM. Protein was determined by Bradford assay. For western blot analysis, SSM and IFM were additionally centrifugated using percoll buffer to increase the purity of preparations. Protein was determined by Bradford assay.

### Studies in isolated mitochondria

#### Respiration assay

O_2_ consumption was quantified in SSM and IFM from young and old mice hearts incubated in respiration buffer (in mmol/l: 100 KCl, 5 MOPS pH 7.4, 2 EGTA, 5 KH_2_PO_4,_ 1 MgCl_2_, 0.1% deffated albumin) using Clark-type oxygen electrodes (Hansatech, Norfolk, UK) after the addition of specific substrates for each of the respiratory complexes (complex 1: 2 mmol/l malate+5 mmol/l glutamate; complex 2: 6 mmol/l succinate with 0.5 *μ*mol/l rotenone to inhibit complex 1; complex 3: reduced form of 2,3 dimethoxy-5-methyl-1,4-benzoquinone; complex 4: ascorbate+NNN′N′ tetramethyl-p-phenylenediamine (TMPD)). Maximal O_2_ consumption (state-3) was obtained with 0.25 mmol/l ADP. RCR was calculated as ADP-stimulated O_2_ consumption (state-3)/non-stimulated O_2_ consumption (state-2). CS activity was determined by colorimetry and was used to normalize data on mitochondrial respiration.^41^

#### Mitochondrial calcium uptake

Mitochondrial Ca^2+^ uptake was fluorometrically quantified in 150 *μ*g of SSM or IFM incubated in 235 *μ*l of respiration buffer with 0.5 *μ*mol/l calcium green-5N (CG5N hexapotassium salt, Invitrogen, Madrid, Spain). Mitochondria were exposed to an external Ca^2+^ pulse of 30 *μ*mol/l in the presence of 1 *μ*mol/l cyclosporine A to avoid membrane permeabilization. Changes in 530 nm fluorescence were recorded every 10 s. Fluorescence decay after Ca^2+^ addition reflected the kinetics of mitochondrial Ca^2+^ uniporter and could be effectively inhibited by 10 *μ*mol/l of the specific blocker Ru360.

### Isolation of mouse cardiac myocytes

For the obtention of cardiomyocytes, hearts from young (<6 months) or old (>20 months) mice were perfused in a Langendorff system (Trallero, Barcelona, Spain) for 15 min with a modified Krebs buffer (in mmol/l: 110 NaCl, 2.6 KCl, 1.2 KH_2_PO_4_, 1.2 MgSO_4_, 11 glucose and 10 butanedione monoxime, pH 7.4) with 0.03% type II collagenase (Serva, Heidelberg, Germany). Perfused tissue was subjected to differential centrifugation. Calcium tolerant rod-shaped cardiomyocytes were selected by sedimentation in 4% BSA (bovine serum albumin) gradient, and plated on laminin-coated cover slips. Only when the initial yield of rod-shaped cardiac myocytes was >50%, preparations were considered suitable for experiments.

### Lysosome, mitochondria and lipofuscin staining

Lysosomes were visualized in cardiomyocytes loaded with 50 nmol/l lysotracker green (30 min, 37 °C) and excited at 560 nm using an Ar/Kr laser confocal system (Yokogawa CSU10, Tokyo, Japan; Nipkow spinning disk), set on an Olympus IX70 (VoxCell Scan, Visitech, Sunderland, UK) with × 60 oil immersion objective lens. Mitochondrial pool was estimated in confocal images of intact cells after labeling mitochondria with 1 *μ*mol/l MitoTracker Red CMXRos (30 min, 37 °C). Lipofuscin pigment was observed as autofluorescence after 488 nm laser excitation. In all cases, cell fluorescence was analyzed in background-substracted images using commercially available software (VoxCell Scan, Visitech).

### Mitochondrial membrane potential (Δ*Ψ*m) in intact cardiomyocytes

Resting Δ*Ψ*m was analyzed in isolated cardiomyocytes loaded with 10 *μ*mol/l JC-1 (6 min, 37 °C) and excited at 488 nm (Ar/Kr laser confocal system). Green (520 nm) and red (590 nm) emission lights were simultaneously recorded at × 60 (CCD cameras, Hamamatsu City, Japan) and 590/520 nm fluorescence ratio was calculated as an index of Δ*Ψ*m and expressed relative-to-maximal mitochondrial membrane depolarization achieved with 200 *μ*mol/l dinitrophenol (DNP).

### Induction of SR–mitochondria calcium transfer in permeabilized cardiomyocytes

To analyze the fraction of mitochondrial Ca^2+^ uptake that is dependent on SR Ca^2+^ transfer without the contribution of sarcolemma/cytosol, cardiomyocytes were loaded with 5 *μ*mol/l rhod-2 using the cold–warm protocol (60 min at 4 °C followed by 30 min at 37 °C) and the sarcolemma of the cells was subsequently permeabilized with 10 *μ*mol/l digitonin (2 min, 37 °C) in intracellular-like buffer (in mmol/l: 5 MgCl2, 10 HEPES, 250 sucrose, 25 Tris, 5 succinate, 2 ATP, pH 7.2) under Ca^2+^ free conditions (2 mmol/l EGTA). SR Ca^2+^ release was induced with a pulse of caffeine (10 mmol/l) and mitochondrial Ca^2+^ uptake was monitored as changes in rhod-2 fluorescence throughout time with respect to the initial value (F/F0, Ex:561 nm/Em:605 nm) using an Ar/Kr laser confocal system (Yokogawa CSU10, Nipkow spinning disk), set on an Olympus IX70 (VoxCell Scan, Visitech) at × 60.

### Quantification of NAD(P)H regeneration

NAD(P)H regeneration from NAD(P) was determined in intact cardiomyocytes as autofluorescence after excitation at 340 nm (Em:450 nm) with the aid of a xenon lamp (Visitech monochromator, UK) on the stage of an inverted microscope (Olympus IX70, Tokyo, Japan), at × 40. For calibration, maximal reduction state of NAD(P)H was achieved after the addition of 2 mmol/l sodium cyanide, and minimal reduction state was achieved after the addition of 200 *μ*mol/l DNP. Results were expressed as the ratio of NADH(P)H/NAD(P)+ and as percentage of the reduced form with respect to total NAD(P)H pool according to the calibrated data.^15^ To test the effect of increasing contractile activity on NAD(P)H consumption/regeneration, electrical pacing of the cells was increased from 1 Hz to 5 Hz.

### Cytosolic and mitochondrial ROS production

ROS production in cytosolic and mitochondrial compartments was analyzed in freshly isolated cardiomyocytes simultaneously labeled with 10 *μ*mol/l of the cytosolic ROS-sensitive fluorochrome 2',7'-dichlorodihydrofluorescein diacetate (30 min, 37 °C) and 5 *μ*mol/l of the mitochondrial ROS-sensitive fluorochrome MitosSOX red (5 *μ*mol/l,10 min, 37 °C). Cells were electrically stimulated at low (1 Hz) and high frequencies (5 Hz). At the end of the protocol, 250 *μ*mol/l of the mitochondrial respiratory complex 1 inhibitor rotenone was added to induce a peak of ROS production. Fluorescence was recorded using an Ar/Kr laser confocal system (Visitech, Ex: 488 nm/Em: 520–580 nm) on the stage of an inverted microscope (Olympus IX70). Results were expressed as relative changes with respect to peak ROS induced by rotenone. Image acquisition was set at 1 image/10 s to avoid phototoxicity.

### SR calcium handling

#### SR Ca^2+^ transients in field-stimulated cardiomyocytes

Intact cardiomyocytes loaded with the cytosolic Ca^2+^ indicador fluo-4 (5 *μ*mol/l, 30 min at 37 °C) were superfused with a control HEPES buffer (NaCl 150 mmol/l, KCl 5.4 mmol/l, HEPES acid 10 mmol/l, CaCl_2_ 2 mmol/l, glucose 1 mmol/l, pyruvate 2.5 mmol/l, creatine 5 mmol/l, taurine 5 mmol/l, at pH=7.4) and electrically stimulated at 1 Hz (biphasic pulse, SIU-102, Warner Instruments, Hamden, CT, USA). Fluorescence changes throughout time were recorded using an Ar/Kr laser confocal system (Yokogawa CSU10, Nipkow spinning disk), set on an Olympus IX70 (VoxCell Scan, Visitech) at × 60, and expressed as changes throughout time with respect to the initial value (F/F0; Ex:488 nm/Em:520 nm). Image acquisition was set at 40 images/s. Data were analyzed with VoxCell Scan software (Visitech).

#### Spontaneous SR Ca^2+^sparks

Spontaneous SR Ca^2+^ sparks were analyzed in intact fluo-4 loaded cardiomyocytes incubated in control HEPES buffer using a spectral confocal microscope (Ex:488 nm, Olympus Spectral Confocal Microscopy FV1000, Olympus). Spark frequency (sparks × (100 *μ*m^−1^) × (s^−1^)), amplitude (ΔF/F_0_), rate (Δ*F*/*F*_0_ × (s^−1^)) and morphology (full width at half-maximal amplitude, *μ*m) were quantified using Spark Master plugin of ImageJ software (NIH, Bethesda, MD, USA).

#### SR Ca^2+^ load

Total SR Ca^2+^ load was analyzed in intact cardiomyocytes loaded with the cytosolic Ca^2+^ indicator fluo-4 (5 *μ*mol/l, 30 min at 37 °C). Cells were submitted to a brief field-stimulation at 1 Hz for 30 s to allow Ca^2+^ transient stabilization followed by a single pulse of 10 mmol/l caffeine. Maximal amplitude of caffeine-induced peak fluorescence was normalized by the initial fluorescence value (F/F0) and considered as an index of total SR Ca^2+^ load.

### Spatial proximity between SR and mitochondria

#### Immunolabeling and colocalization of RyR and VDAC

Isolated cardiomyocytes placed on laminin-coated coverslips were fixed (99.6% acetone cooled at −20 °C, 5 min), permeabilized (0.025% Triton X-100 in phosphate-buffered saline (PBS)) and incubated at 4 °C overnight with mouse monoclonal RyR antibody (34C Abcam, Cambridge, UK; 1 : 50) and rabbit polyclonal VDAC antibody (15895Abcam, 1 : 50) in PBS–BSA 1%. This was followed by 1 h incubation at room temperature with secondary Alexa antimouse-561 and Alexa antirabbit-488. Nuclei were stained with 10 *μ*g/ml Hoeschst-33342. Mounted samples were observed with a spectral confocal microscope (Olympus Spectral Confocal Microscopy FV1000, Olympus). Degree of RyR-VDAC overlap was quantified with Mander's coefficient analysis (JaCop, ImageJ software).

#### Proximity ligation assay

Isolated cardiomyocytes were simultaneously labeled with monoclonal RyR antibody (34C Abcam, 1 : 50) and rabbit polyclonal VDAC antibody (15895Abcam, 1 : 50), followed by the corresponding secondary antibodies (Alexa antimouse-561 and Alexa antirabbit-488) conjugated with the PLA oligonucleotide probes (PLA probe rabbit PLUS and PLA probe rat MINUS; Olink Bioscience, Uppsala, Sweden) for 1 h at 37 °C. For ligation and amplification reactions, Duolink InSitu detection kit recommendations were followed (Olink Bioscience). Cell images were registered with a spectral confocal microscope (FluoView-1000, Olympus). Positive fluorescent cross-reactivity was quantified in background-substracted images using BlobFinder software (Centre for Image Analysis, Uppsala University, Sweden).

### Western blot

Mouse heart homogenates, microsomal, MAMs and mitochondria extracts were obtained by protein fractionation. Briefly, fresh cardiac mouse ventricles were minced and homogenized using a Potter–Elvehjem PTFE pestle-glass tube at 1400 r.p.m. on ice-cold isolation buffer (HEPES acid 10 mmol/l, manitol 225 mmol/l, sucrose 75 mmol/l, EGTA 0.1 mmol/l at pH=7.4). After an initial centrifugation step (750 × *g* for 5 min, 4 °C), nuclei and other cell debris were discarded in the pellet, and supernatant was centrifuged (10 000 × *g* for 10 min, at 4 °C). The resulting pellet contained crude mitochondrial fraction. Supernatant was ultracentrifuged (100 000 × g for 1 h, at 4 °C) to obtain cytosolic fraction in the supernatant and microsomal vesicles in the pellet. Crude mitochondrial fraction previously obtained was purified in a 30% Percoll gradient column. Percoll gradient column was ultracentrifuged (90 000 × *g*, for 30 min at 4 °C) in a swinging rotor, resulting in pure mitochondria (heavy fraction) and MAM (light fraction).^42^ Equal amounts of protein (70 *μ*g) supplemented with 1% SDS (Bio-Rad, Hercules, CA, USA; 161–0301) were subjected to PAGE. Samples were preheated (95 °C, 5 min) in SDS reducing buffer, separated on 6–12% acrylamide/bis (Bio-Rad) gels and transferred to Amersham Hybond-ECL nitrocellulose membranes (GE Healthcare). The membranes were blocked in TBS-T solution (Tris-buffered saline, 0.1% Tween-20) with 5% non-fat milk powder. Proteins were detected with RyR (Abcam, ab2868), VDAC1 (Abcam, ab15895), GRP75 (SantaCruz Biotechnology Inc., Dallas, TX, USA; sc-13967), GAPDH (Gene Tex, San Antonio, TX, USA; GT239), Mitofusin-2 (Abcam, ab56889) and ANT1/2 (SantaCruz Biotechnology Inc., sc-9299) antibodies in TBS-T with 3% BSA (Sigma). Horseradish peroxidase-conjugated IgGs were used as secondary antibodies: antimouse (Sigma, A4416), antirabbit (Pierce, 31460), and anti-goat (Thermo Scientific, Waltham, MA, USA; 31402). Peroxidase reactions were carried out and visualized using Supersignal West Dura Extended Duration Substrate (Thermo Scientific) and the chemiluminescence imaging system LAS-3000 (Fujifilm, Tokyo, Japan). Band intensities were quantified with Science Lab-2001 Image Gauge (Fujifilm).

### *β*-gal quantification in mouse hearts

*β*-gal was determined in heart extracts from young and old mice using the soluble *β*-galactosidase method.^43^ Frozen tissue (0.1–0.2 g) was homogenized in 0.1 mol/l citrate (pH 4.5) and centrifuged at 12 000 × *g* for 7 min. The supernatants were diluted in citrate assay buffer containing 2-nitrophenyl-*β*-D-galactopyranoside (2 mg/ml) and 1 mmol/l MgCl_2_. After overnight incubation at 37 °C, one volume of 1 mol/l potassium carbonate was added at each sample, and absorbance of *o*-nitrophenol, the product resulting from the enzyme activity, was read at 420 nm (E_mM pH10_ 21.3 ). *β*-gal was expressed as μmols of *o*-nitrophenol/g tissue.

### Measurement of GSH levels in mouse hearts

GSHtot concentration and oxidized GSH fraction (GSSG) were determined in heart extracts from young and old animals using the GSH reductase enzymatic method,^44^ based on the reaction between Ellman's reagent (DTNB) with GSH to form a spectrophotometric detectable product at 412 nm. A standard curve of reduced GSh (0–50 mM) was used to calculate GSH amounts in mice extracts. Briefly, frozen heart extracts from old and young animals were thawed (50 *μ*l) and subsequently assessed in phosphate-EDTA buffer containing GSH reductase (17 U/ml), NADPH (0.016 mg/ml) and DTNB (0.042 mg/ml). To determine GSSG, a duplicate of each sample was previously incubated with 3 mmol/l of 1-methyl-2-vinylpyridinium triflate, a thiol-scavenging reagent, which rapidly scavenges reduced form of GSH without interfering with the enzyme activity. Results were expressed in nmol GSH/mg protein.

### Differential quantitative mitochondrial proteomic analysis

Mitochondrial and microsomal fractions were isolated by differential centrifugation (as described above). Peptides and proteins were identified and quantified by differential high-throughput proteomic analysis performed by stable isotopic labeling using a previously described protocol.^45, 46, 47^ Changes in the abundance and composition of cysteine-thiol redox status of mitocondria and microsomal proteins were quantified using the GELSILOX methodology.^48^ Analyses of samples by LC-MS/MS were performed as previously described.^49^ Functional protein classification was done using the Gene Ontology database. Proteomics results were analyzed as previously described.^50^

### Statistical analysis

Data are expressed as mean±S.E.M. For comparisons between two groups two-tailed Student's *t*-test for independent samples was used. For comparison of groups with more than one factor, a factorial ANOVA analysis was performed followed by *post hoc* comparisons when necessary. Differences of *P*<0.05 were considered statistically significant. When samples did not follow a normal distribution, the non-parametric test of median was used. All statistical analyses were performed with SPSS v.15 software (New York, NY, USA).

## Figures and Tables

**Table 1:** Echocardiographic study of young and old mice

	**Young (*n*=12)**	**Old (*n*=14)**	***P***
	**Mean±S.E.M.**	**Mean±S.E.M.**	
Body weight (g)	38.30±4.00	34.59±1.90	0.41
SWT (mm)	0.87±0.05	0.79±0.021	0.19
PWT (mm)	0.88±0.04	0.72±0.029	0.004*
LVEDD (mm)	4.09±0.17	4.30±0.174	0.39
LVEDDIN (mm/g)	0.12±0.01	0.13±0.009	0.17
PWT/LVEDD	0.22±0.01	0.17±0.01	0.016*
SWT/LVEDD	3.13±0.2	3.75±0.25	0.07
LVm (mg)	115.42±11.89	100.98±6.25	0.25
EDLvol (mL)	46.98±4.1	55.12±4.29	0.37
LVm/EDLvol (mg/mL)	3.32±0.15	1.88±0.08	0.014*
LVEF (%)	71.01±2.73	66.85±2.06	0.22
HR (b.p.m.)	455.45±9.75	436.36±3.24	0.32

EDLVvol, end-diastolic left ventricular volume; HR, heart rate; LVEDD, left ventricular end-diastolic diameter; LVEDDIN, LVEDD indexed by weight; LVEF, left ventricular ejection fraction; LVm, left ventricular mass; PWT, posterior wall thickness; SWT, septum wall thickness; b.p.m., beats per minute

## Abbreviations

a.u.: arbitrary units
a.u.f.: arbitrary units of fluorescence
CS: citrate synthase
DNP: dinitrophenol
GSH: glutathione
GSSG: glutathione disulfide
IFM: interfibrillar mitochondria
LVEF: left ventricular ejection fraction
LVEDD: left ventricular end-diastolic diameter
LVESD: left ventricular end-systolic diameter
MAM: mitochondria-associated membranes
PLA: proximity ligation assay
PWT: posterior wall thickness
RCR: respiratory control rate
RyR: ryanodine receptor
SSM: subsarcolemmal mitochondria
SWT: septum wall thickness
TBS: tris-buffered saline
VDAC: voltage-dependent anion channel
