# Supplementary material for: Defective sarcoplasmic reticulum–mitochondria calcium exchange in aged mouse myocardium
Source: Cell Death Dis. 2014 Dec 18;5(12):e1573–. doi: 10.1038/cddis.2014.526 (PMC4454162; doi:10.1038/cddis.2014.526)
Supplement: Supplementary Information [file cddis2014526x3.doc]

**Figure Legends for Supplementary Tables**

**Supplementary Table 1:** Changes in relative abundance of proteins of oxidative phosphorylation complexes in subsarcolemmal (SSM) and interfibrillar mitochondria (IFM) isolated from aged mouse hearts respect to young mouse hearts.

Negative Zq values mean that the protein is increased and positive Zq values that the protein is decreased.

**Supplementary Table 2:** List of identified mitochondrial peptides containing oxidized and reduced cysteine residues in subsarcolemmal (SSM) and interfibrillar mitochondria (IFM) from mouse hearts.
